# New Faces of old Friends: Emerging new Roles of RNA-Binding Proteins in the DNA Double-Strand Break Response

**DOI:** 10.3389/fmolb.2021.668821

**Published:** 2021-05-07

**Authors:** Julie A. Klaric, Stas Wüst, Stephanie Panier

**Affiliations:** ^1^ Max Planck Institute for Biology of Ageing, Cologne, Germany; ^2^ Cologne Cluster of Excellence in Cellular Stress Responses in Aging-Associated Diseases (CECAD) Research Center, University of Cologne, Cologne, Germany

**Keywords:** DNA double strand break, RNA-binding protein, genome stability, DNA repair, non-coding RNA, phase separation, DSB response, DNA damage

## Abstract

DNA double-strand breaks (DSBs) are highly cytotoxic DNA lesions. To protect genomic stability and ensure cell homeostasis, cells mount a complex signaling-based response that not only coordinates the repair of the broken DNA strand but also activates cell cycle checkpoints and, if necessary, induces cell death. The last decade has seen a flurry of studies that have identified RNA-binding proteins (RBPs) as novel regulators of the DSB response. While many of these RBPs have well-characterized roles in gene expression, it is becoming increasingly clear that they also have non-canonical functions in the DSB response that go well beyond transcription, splicing and mRNA processing. Here, we review the current understanding of how RBPs are integrated into the cellular response to DSBs and describe how these proteins directly participate in signal transduction, amplification and repair at damaged chromatin. In addition, we discuss the implications of an RBP-mediated DSB response for genome instability and age-associated diseases such as cancer and neurodegeneration.

## Introduction

DNA double-strand breaks (DSBs) are highly deleterious DNA lesions that occur as a consequence of unresolved replication stress or after exposure to certain chemicals or to ionizing radiation. In addition, they are formed in a programmed manner during meiosis and during antibody diversification (Keeney et al., 2014; Methot and Di Noia, 2017). Un- or misrepaired DSBs lead to the accumulation of gross chromosomal rearrangements and mutations that cause loss of genetic information. As such, they are potent inducers of genome instability and threaten cellular function and cell survival. Cells respond to DSBs by activating a multi-layered signaling and repair network (known as the DSB response) that integrates many cellular processes including the hierarchical assembly of signaling and repair factors at DSB sites, local chromatin remodeling, cell cycle arrest and, eventually, DNA repair. The importance of a functional DSB response is underscored by the fact that defects in DSB repair contribute to the etiology of numerous diseases including premature aging, neurodegeneration and cancer (Tiwari and Wilson, 2019).

**FIGURE 1:**
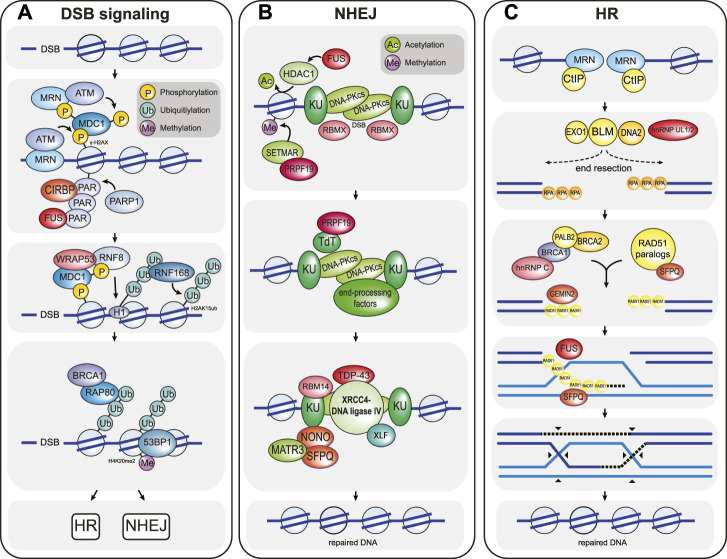
Overview of the chromatin-based DSB response. **(A)** DSB signaling is initiated by the MRN complex, which senses DSBs and recruits the ATM kinase to the damaged chromatin. Here, ATM phosphorylates the histone H2A variant H2A.X to form γ-H2AX, which in turn is recognized by MDC1. MDC1 is constitutively phosphorylated by CK2 (not shown) and activates a positive feedback loop by recruiting more MRN and ATM to the break site. In addition, MDC1 is phosphorylated by ATM, and this phosphorylation event triggers the recruitment of the E3 ubiquitin ligase RNF8. RNF8 in turn ubiquitylates histone H1, which is then bound by a second E3 ubiquitin ligase called RNF168. RNF168 ubiquitylates histone H2A at residues K13/K15 (H2AubK13/15). The resulting ubiquitin signal is bound by multiple proteins, including RNF168 itself (to create a second positive feedback loop that locally amplifies the DNA damage signal) and the DSB repair pathway choice proteins BRCA1 and 53BP1. BRCA1 recognizes ubiquitylated chromatin via its interaction partner RAP80 and promotes HR-mediated DSB repair. In contrast, 53BP1is enriched at damaged chromatin by simultaneously binding to RN168-ubiquitylated H2A and to constitutively methylated histone H4K20me2, and promotes NHEJ-mediated DSB repair. CIRBP, WRAP53, and FUS are examples of RBPs that modulate DSB signaling. CIRBP enhances the accumulation of MRN-ATM at the DSB site, while WRAP53 facilitates RNF8 recruitment by stabilizing the MDC1-RNF8 interaction. FUS likely promotes the retention of signaling and repair factors by participating in phase separation around the break DSB site. **(B)** NHEJ-mediated DSB repair is initiated when DSB ends are recognized by the KU proteins, followed by recruitment of the kinase DNA-PKcs. KU-DNA-PKcs mostly act as a scaffold to recruit numerous DNA end-processing factors (including TdT and many others such as DNA polymerases λ and μ, Artemis, PNKP, and TDP1; not shown) that create DNA ends compatible for re-ligation. Following DNA end-processing, the DNA ligation complex XLF-XRCC4-DNA ligase IV is recruited to the DSB to re-join the broken DNA ends. Numerous RBPs promote NHEJ. For example, FUS enhances NHEJ by recruiting the histone deacetylase HDAC1 and as such enhancing local chromatin remodeling. RBMX binds to DSB ends to prevent DNA end-resection. PRPF19 recruits the histone methyltransferase SETMAR, which enhances the recruitment of NHEJ factors such as KU, and also binds to the DNA end-processing factor TdT. TDP43, RBM14, and SFPQ-NONO act as scaffolds that stabilize the assembly of the DNA ligation complex. **(C)** HR-directed DSB repair is initiated by extensive DNA end-resection by the nucleases CtIP, MRE11 (which is part of the MRN complex), EXO1 and DNA2-BLM. DNA end-resection generates long single-stranded DNA overhangs that are rapidly bound by RPA. Next, with the help of numerous mediator complexes, including the BRCA2-PALB2-BRCA1 and the RAD51 paralog complexes, RPA is exchanged for the DNA recombinase RAD51. The resulting RAD51 nucleoprotein filament then searches for homologous sequences elsewhere in the genome (usually the sister chromatid), where it then catalyzes strand invasion to form a DNA crossover called a Holliday junction. This generates a primer for DNA synthesis (dashed lines), which is extended by branch migration away from the crossover site. After DNA repair, the joint DNA molecules are resolved by cleavage of the crossed or non-crossed DNA strands (black arrowheads). The RBPs hnRNP UL 1 and 2 enhance DNA end-resection by stimulating BLM recruitment. hnRNPC, GEMIN2 and SFPQ support the formation of the RAD51 nucleoprotein filament. In addition, GEMIN2, SFPQ, and FUS also stimulate strand exchange. Note that DSB signaling and repair reaction require many additional factors, posttranslational modifications and various species of local non-coding RNAs that are not depicted. Selected RBPs that participate in the chromatin-based response are highlighted in red in all three panels. Additional RBPs that participate in DSB signal transduction and repair are listed in Table 1.

The signaling pathway that is mounted in response to DSBs activates one of two main repair pathways, the choice of which depends largely on the cell cycle phase and on the presence of homologous sequence that can be used as a repair template (Figure 1A). DSB repair that uses very little homologous sequence is termed non-homologous end-joining (NHEJ) and typically involves several iterative steps of DNA end-processing followed by ligation of the DNA ends (Figure 1B) (Pannunzio et al., 2018). It is active throughout the cell cycle but is especially relevant during the G0 and G1 phases. During NHEJ, DSB ends are first recognized and bound by the KU 70/80 heterodimer, which protects the ends from extensive DNA end-resection and acts as a scaffold to assemble downstream NHEJ factors at the break site, most notably nucleases (DNA-PKcs-Artemis, APLF), DNA polymerases (DNA Pol λ, DNA Pol μ, TdT) and the DNA ligation complex (XLF-XRCC4-DNA ligase IV) (Figure 1). Chemical modifications or mismatching overhangs at the broken DNA ends often prevent direct re-ligation by XLF-XRCC4-DNA ligase IV, which explains the need to recruit end-processing enzymes in the form of nucleases and polymerases to generate ends that are compatible with ligation. The processing of DSB ends can result in the addition or loss of nucleotides and as such allows a certain degree of genetic variability, which is essential for physiological processes such as V(D)J and class switch recombination (Bétermier et al., 2014; Pannunzio et al., 2018). In contrast, homologous recombination (HR) is generally a high-fidelity repair pathway because it utilizes long stretches of homologous sequence as a template for repair (Figure 1C) (Lisby and Rothstein, 2015). It only operates in the late S and G2 phases of the cell cycle when the sister chromatid is available as a repair template. HR-directed repair of a DSB is initiated by the extensive resection of the DNA ends by the nucleases CtIP, MRE11, EXO1, and DNA2-BLM. DNA end-resection generates long 3’ single-stranded DNA (ssDNA) overhangs, which are rapidly recognized and bound by RPA. During the next step of HR, the single-stranded DNA-bound RPA is exchanged for the recombinase enzyme RAD51 and the resulting RAD51-ssDNA filaments search for homologous sequences elsewhere in the genome. RAD51 then catalyzes strand invasion and the formation of a Holliday junction. This is followed by DNA synthesis and processing of the joint DNA molecules to complete DNA repair (Figure 1C) (Lisby and Rothstein, 2015).

**TABLE 1:** List of human RNA-binding proteins that participate in the chromatin-based DSB response.

RNA-binding protein	Functions in the DSB response	Selected references
53BP1	NHEJ and phase separation	Pryde et al. (2005), Kilic et al. (2019), Pessina et al. (2019)
Argonaute-2	HR and recruitment of diRNAs	Wei et al. (2012), Gao et al. (2014)
BRCA1	HR	Sharma et al. (2015)
CIRBP (hnRNP A18)	DSB signaling	Chen et al (2018)
WRAP53	DSB signaling	Henriksson et al. (2014), Rassoolzadeh et al. (2016)
DDX1	RNA: DNA hybrid resolution	Li et al. (2008), Li et al. (2016)
DDX5	RNA: DNA hybrid resolution	Yu et al. (2020), Sessa et al. (2021)
DDX17	Suppressor of HR	Adamson et al. (2012)
DNA-PKcs	NHEJ	Zhang et al. (2016)
DNA polymerase ζ (yeast)	RNA-templated DSB repair	Meers et al. (2020)
Drosha	DDRNA and diRNA production	Francia et al. (2012), Lu et al. (2018)
Dicer	DDRNA production	Francia et al. (2012)
EDC4	HR	Hernández et al. (2018)
EWS	Phase separation	Altmeyer et al. (2015)
EXOSC10	DDRNA removal and HR	Marin-Vicente et al. (2015)
FUS (hnRNP P2)	DSB signaling, HR, NHEJ, and phase separation	Baechtold et al. (1999), Bertrand et al. (1999); Wang et al. (2013), Altmeyer et al. (2015)
GEMIN2	HR	Takizawa et al. (2010), Takaku et al. (2011)
hnRNP B1	Negative regulator of NHEJ	Iwanaga et al. (2005)
hnRNP C	HR	Anantha et al. (2013)
hnRNP D	RNA: DNA hybrid resolution and HR	Alfano et al., (2019)
hnRNP U	NHEJ	Hegde et al. (2016)
hnRNP UL1	HR	Polo et al. (2012), Hong et al. (2013)
hnRNP UL2	HR	Polo et al. (2012), Hong et al. (2013)
KU	NHEJ	Yoo and Dynan (1998)
METTL3	Promotes RNA: DNA hybrid formation and HR	Zhang et al. (2020)
NONO	NHEJ	Li et al. (2009), Krietsch et al. (2012)
PRPF19	NHEJ	Mahajan and Mitchell (2003), Beck et al. (2008)
RAD52	HR and RNA-templated DSB repair	Keskin et al. (2014)
RBM14	NHEJ and generation of RNA: DNA hybrids	Simon et al. (2017), Jang et al. (2020)
RBMX (hnRNP G)	NHEJ	Shin et al. (2007), Adamson et al., (2012)
RNase H2	RNA: DNA hybrid resolution	D’Alessandro et al. (2018)
RNAP III	HR	Liu et al. (2021)
RPA	RNA-templated DSB repair	Kim et al. (1992), Mazina et al. (2017)
Senataxin	RNA: DNA hybrid resolution	Cohen et al. (2018)
SFPQ	HR and NHEJ	Udayakumar et al. (2003), Bladen et al., (2005), Morozumi et al. (2009), Rajesh et al. (2011)
TAF15	Phase separation	Altmeyer et al. (2015)
TDP-43	NHEJ and R-loop prevention	Hill et al. (2016), Mitra et al. (2019)
XPG	RNA: DNA hybrid resolution	Yasuhara et al. (2018)
XRN2	RNA: DNA hybrid resolution	Morales et al. (2016), Dang and Morales (2020)

DSB-induced signaling and the downstream DSB repair pathways rely heavily on protein-protein interactions, which are often mediated by damage-induced post-translational modifications (PTMs) such as poly (ADP-ribosyl)ation, phosphorylation, ubiquitylation, SUMOylation and acetylation (Figure 1) (Matsuoka et al., 2007; Bennetzen et al., 2010; Elia et al., 2015; Morris and Garvin, 2017). The interplay of these PTMs at damaged chromatin is highly complex and forms the backbone of a timely and efficient DSB response (Dantuma and van Attikum, 2016). Besides PTMs, RNAs and their interactions with both DNA and proteins have emerged as central regulatory elements of DSB signaling and repair. While it has long been known that the processing and transport of protein-coding mRNAs is crucial for the DSB response (Wickramasinghe and Venkitaraman, 2016), long and short non-coding RNAs have only recently been identified as direct regulators of this pathway. Indeed, the last years have seen the identification of an ever-growing network of diverse non-coding RNA species that locally modulate DSB signaling and repair (Mikolaskova et al., 2018; Bader et al., 2020; Ketley and Gullerova, 2020). Not surprisingly, the RNA network surrounding the DSB response is tightly associated with RNA-binding proteins (RBPs), many of which are themselves subject to DNA damage-induced PTMs (Matsuoka et al., 2007; Paulsen et al., 2009; Bensimon et al., 2010; Hurov et al., 2010; Słabicki et al., 2010; Adamson et al., 2012; Beli et al., 2012; Izhar et al., 2015; Shkreta and Chabot, 2015).

The human genome encodes well over 1,500 RBPs, which can be classified into over 1,000 distinct families (Gerstberger et al., 2014). Many of the RBPs that function in the DSB response are members of the hnRNP (heterogeneous ribonucleoprotein particle), DEAD-box helicase, FET (FUS/TLS, EWS, and TAF15), and DBHS (*Drosophila* behavior human splicing) families of RNA-binding proteins (Haley et al., 2009; Altmeyer et al., 2015; Knott et al., 2016; Bader et al., 2020). RBPs are functionally highly versatile proteins that achieve binding specificity through the action of modular RNA-binding domains (Hentze et al., 2018). Recently, it was estimated that the RNA-binding proteome contains approximately 600 structurally distinct canonical and non-canonical RNA-binding domains (Gerstberger et al., 2014). Canonical RNA-binding motifs include the RNA recognition motif (RRM), the K-homology domain (KH), and the RNA-binding domain consisting of Arg-Gly-Gly repeats (RGG), all of which can be found in the major RBP families. Interestingly, numerous RBPs are devoid of such canonical RNA-binding domains and instead bind to RNA in a non-conventional fashion, for example via so-called intrinsically disordered regions (IDRs) (Hentze et al., 2018).

**FIGURE 2:**
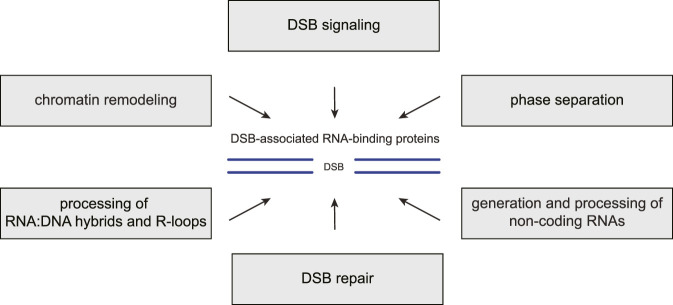
RBP functions in the chromatin-based DSB response. Many RNA-binding proteins (RBPs) associate with DNA double-strand breaks (DSBs) and the surrounding chromatin where they carry out several distinct functions. First, they promote the recruitment or modulate the activities of DSB signaling and repair factors. Second, they regulate local chromatin remodeling. Third, they contribute to liquid-liquid phase separation around the break site. Fourth, they prevent, resolve or stabilize RNA:DNA hybrids and R-loops. Fifth, they participate in the generation or processing of various non-coding RNA species at DSB sites.

It is well established that cells adjust their gene expression profiles to accommodate an efficient DSB response, and a considerable number of RBPs have been shown to regulate the expression of signaling and repair proteins following DSB formation (Wickramasinghe and Venkitaraman, 2016; Mikolaskova et al., 2018). However, it is becoming increasingly clear that many DSB response-associated RBPs have additional functions in signaling and repair that are unrelated to their canonical functions in gene expression and splicing, particularly locally at the chromatin surrounding DSBs (Table 1). Here, we review these emerging new roles of RBPs in the DSB response (Figure 2). We highlight key examples of how RBPs participate in different DSB signaling and repair steps, and we discuss their roles in regulating RNA metabolism and phase separation locally at the chromatin surrounding DSB sites.

## RNA-Binding Proteins Participate in Double-Strand Break Signaling

DSB formation triggers the rapid recruitment of a large plethora of proteins to the break site. This local concentration of proteins amplifies and transduces the DNA damage signal to the rest of the nucleus and makes the DSB and its surrounding chromatin competent for repair. The recruitment of signaling and repair factors is highly dynamic, and the total residence time on damaged chromatin can last from seconds to hours, depending on protein function (Bekker-Jensen et al., 2006). Numerous RBPs have been shown to accumulate at damaged chromatin, often in a PARP1-dependent manner and within seconds of DSB formation, suggesting direct functions in DSB signaling or repair (Adamson et al., 2012; Krietsch et al., 2012; Hong et al., 2013; Mastrocola et al., 2013; Izhar et al., 2015; Chen et al., 2018; Jang et al., 2020).

An RBP that localizes to DSB sites particularly early in the response is CIRBP (Cold-inducible RNA-binding protein, also known as hnRNP A18). It is part of the hnRNP family of RNA-binding proteins, which regulate many aspects of RNA metabolism including mRNA maturation, stabilization, transport and translation (Geuens et al., 2016). Indeed, CIRBP, which is upregulated after genotoxic stress, is best known as a translational activator of stress-responsive transcripts such as RPA2 and ATR (Yang and Carrier, 2001; Yang et al., 2010). However, a recent study has demonstrated that CIRBP is also rapidly recruited to DSB sites in a PARP1-dependent manner where it promotes DSB signaling by enhancing the chromatin recruitment of the MRN complex and of ATM. Accordingly, cells depleted of CIRBP display reduced DNA repair by both HR and NHEJ. Interestingly, upon DSB recruitment, CIRBP is poly (ADPribosyl)ated and then rapidly excluded again from the damaged chromatin (Chen et al., 2018). Similar association and dissociation dynamics have been observed for other RBPs as well (Salton et al., 2010; Ha et al., 2011; Adamson et al., 2012; Beli et al., 2012; Polo et al., 2012; Britton et al., 2014; Altmeyer et al., 2015; Yu et al., 2020; Sessa et al., 2021). Another RBP that is recruited to damaged chromatin upon DSB formation and that appears to directly regulate DSB signaling is WRAP53 (WD40-encoding RNA antisense to p53, also known as TCAB1). The *WRAP53* gene was originally shown to encode an antisense transcript that stabilizes the tumor suppressor p53 in response to DNA damage (Mahmoudi et al., 2009). In addition, *WRAP53* encodes a protein that acts as a scaffolding protein in nuclear membrane-less organelles known as Cajal bodies, which locally concentrate mRNA processing factors and also telomerase (Henriksson and Farnebo, 2015). The importance of WRAP53 for telomerase-mediated telomere maintenance is underscored by the fact that mutations in the *WRAP53* gene cause the two related telomeropathies, dyskeratosis congenita and Hoyeraal–Hreidarsson syndrome (Batista et al., 2011; Zhong et al., 2011; Bergstrand et al., 2020). WRAP53 localizes to DSBs downstream of MDC1 and facilitates the recruitment of the E3 ubiquitin ligase RNF8 to MDC1 by simultaneously binding to both signaling proteins via its WD40 domain (Henriksson et al., 2014). Depletion of WRAP53 impairs RNF8-mediated chromatin ubiquitylation and the downstream recruitment of signaling and repair factors including BRCA1 and 53BP1. Both HR and NHEJ efficiencies are reduced after WRAP53 loss (Henriksson et al., 2014; Rassoolzadeh et al., 2016).

Finally, an RBP that is potentially involved in both DSB signaling and repair is the hnRNP FUS (fused in sarcoma, also known as TLS and hnRNP P2), which has pleiotropic functions in transcription, RNA metabolism, and genome maintenance. The importance of FUS for cell homeostasis is underscored by the fact that variations in the gene encoding FUS have been causatively linked to the development of severe neurodegenerative diseases, particularly amyotrophic lateral sclerosis (ALS) (Deng et al., 2014). Cells lacking FUS are sensitive to ionizing radiation, display a high degree of chromosomal instability and display reduced HR and NHEJ efficiencies (Hicks et al., 2000; Kuroda, 2000; Mastrocola et al., 2013; Wang et al., 2013). In response to DNA damage, FUS is rapidly recruited to damaged chromatin in a PARP1-dependent manner upstream of histone H2A.X phosphorylation (Mastrocola et al., 2013; Wang et al., 2013; Rulten et al., 2014; Aleksandrov et al., 2018; Singatulina et al., 2019). FUS binds directly to poly (ADP-ribose) chains via the same motif that also mediates RNA binding, and it has been proposed that DNA damage-induced poly (ADP-ribose) chains may compete with RNA for FUS binding to locally induce phase separation and as such concentrate DSB signaling and repair factors (see phase separation section below; Mastrocola et al., 2013; Altmeyer et al., 2015). Like CIRBP, FUS association with DSB sites is highly dynamic and the protein is excluded again from damaged chromatin within minutes following the DNA insult (Britton et al., 2014; Altmeyer et al., 2015). Interestingly, FUS is rapidly phosphorylated by both ATM and DNA-PKcs following DNA damage induction (Gardiner et al., 2008; Deng et al., 2014). While the significance of the ATM-mediated phosphorylation event remains unclear, DNA-PKcs-dependent phosphorylation of FUS at its N-terminus changes its phase separation properties and induces its translocation from the nucleus to the cytoplasm in neurons. This translocation effectively removes FUS from the chromatin-based DSB response and instead induces the potentially pathologic accumulation of FUS protein aggregates (Deng et al., 2014; Naumann et al., 2018; Pessina et al., 2020).

## RNA-Binding Proteins Promote Double-Strand Break Repair via Non-homologous End-Joining

While CIRBP and WRAP53 appear to influence the DSB response at the signaling level, a surprisingly large number of RBPs directly affect different steps of NHEJ and HR-directed DSB repair. In the case of NHEJ, RBPs have been suggested to act at the level of KU recruitment, DNA-PKcs autophosphorylation, DNA end synapsis, and the assembly and recruitment of the XRCC4-DNA ligase IV complex. For example, in addition to its function in phase separation, FUS facilitates NHEJ by recruiting the histone deacetylase HDAC1 (histone deacetylase 1) to DSBs, which promotes the local hypoacetylation of H3K53 and is critical for DNA end-joining (Miller et al., 2010; Dobbin et al., 2013; Wang et al., 2013).

Another prominent example of an RBP that directly promotes NHEJ-mediated DSB repair is the hnRNP RBMX (RNA-binding motif protein, X chromosome; also known as hnRNP G). RBMX is a well-known regulator of genome stability that controls splicing and other aspects of mRNA processing of DNA damage response-relevant genes, promotes ATR activation during the replication stress response, and also contributes to mitotic progression and sister chromatid cohesion (Adamson et al., 2012; Matsunaga et al., 2012; Cho et al., 2018; Zheng et al., 2020). Because of its many functions in genome maintenance, it is not surprising that loss of RBMX sensitizes cells to a range of genotoxic agents, including DSB-inducing ionizing radiation. Importantly, following DSB formation, RBMX rapidly localizes to DSBs in a PARP1-dependent manner where it promotes NHEJ-mediated repair by binding to double-stranded DNA (dsDNA) ends, potentially protecting them from the action of exonucleases (Shin et al., 2007; Adamson et al., 2012). Like FUS and other DSB-associated RBPs, it is then quickly released and excluded from the chromatin surrounding the break (Adamson et al., 2012).

A third example of a NHEJ-mediated RBP is PRPF19 (Pre-mRNA-processing factor 19, also known as PSO4). This protein is a key component of multiple subcomplexes that regulate many cellular processes including transcription, splicing and senescence. In addition, it has also long been implicated in diverse genome maintenance pathways such as DNA interstrand crosslink-, DSB- and transcription-coupled repair and in the replication stress response (Chanarat and Sträßer, 2013). Indeed, human PRPF19 was first identified in *S. cerevisiae* as Pso4, whose loss sensitizes yeast to several genotoxins, particularly to DNA interstrand crosslink-inducing agents (Henriques et al., 1989; Rodrigues de Andrade et al., 1989; da Silva et al., 1995; Gray et al., 1996; Revers et al., 2002). In addition, mammalian cells deficient of PRPF19 display a pronounced sensitivity to DSB-inducing agents such as ionizing radiation and etoposide (Mahajan and Mitchell, 2003; Beck et al., 2008; Abbas et al., 2014). PRPF19 is an RNA-binding U-box-type E3 ubiquitin ligase that requires its interaction partner PLRG1 to stimulate its ligase activity (Hildebrandt et al., 2017; de Moura et al., 2018). PRPF19 promotes NHEJ-mediated DSB repair by interacting with and facilitating the recruitment of the histone methyltransferase SETMAR (also known as metnase) to DSBs (Beck et al., 2008; Beck et al., 2010; Beck et al., 2011). At DSBs, SETMAR catalyzes the dimethylation of H3K36 (to form H3K36me2), which promotes the association of early NHEJ factors such as KU70 (Fnu et al., 2011). In addition, PRPF19 interacts with TdT (terminal deoxynucleotidyl transferase), a DNA end-processing enzyme that plays an important role in the NHEJ-mediated repair of DSBs during V(D)J recombination (Mahajan and Mitchell, 2003). Whether and how the RNA-binding and E3 ubiquitin ligase activities of PRPF19 contribute to PRPF19 function during NHEJ remains unclear. Interestingly, PRPF19 also participates in the HR-directed DNA repair of stalled or collapsed replication forks. In response to replication stress, PRPF19 binds to ssDNA-bound phosphorylated RPA via its seven WD repeats and then, together with the E3 ubiquitin ligase RFWD3, ubiquitylates RPA (Maréchal et al., 2014; Wan and Huang, 2014; Dubois et al., 2017). Ubiquitylated RPA in turn is bound by ATRIP, which activates the ATR kinase and induces the HR-directed rescue of stalled or collapsed replication forks (Maréchal et al., 2014; Wan and Huang, 2014; Dubois et al., 2017). Whether PRPF19 also contributes to the HR-directed repair of DSBs outside of the replication stress response is unknown.

TDP-43 (TAR DNA-binding protein 43), another hnRNP, which, similar to FUS, is heavily implicated in the etiopathology of several neurodegenerative diseases including ALS and Alzheimer’s disease (Gao et al., 2018), also plays an important role in NHEJ-mediated DSB repair (Mitra et al., 2019). TDP-43 is canonically involved in alternative splicing and other aspects of mRNA processing, and it also promotes Drosha complex-mediated microRNA biogenesis (Buratti and Baralle, 2008; Kawahara and Mieda-Sato, 2012). Upon DSB formation, TDP-43 rapidly accumulates at damaged chromatin. Here, it promotes NHEJ-mediated repair by acting as a scaffold that helps to recruit the XRCC4-DNA ligase IV complex to the break site (Mitra et al., 2019).

The hnRNP RBM14 (RNA-binding protein 14), which like many other RBPs canonically regulates transcription and splicing, also directly facilitates NHEJ-mediated DSB repair by promoting the autophosphorylation of DNA-PKcs and by helping to recruit the XRCC4-DNA-ligase IV complex to DSB sites, similar to TDP-43 (Auboeuf et al., 2004; Yuan et al., 2014; Simon et al., 2017). It is recruited to sites of DNA damage in a PARP-dependent manner (Jang et al., 2020). Interestingly, RBM14 recruitment also requires its RNA-binding motif, which binds to damage-induced long non-coding RNAs (dilncRNAs) that are transcribed by RNA polymerase II (RNAP II) (Michelini et al., 2017; Jang et al., 2020). Because RBM14 directly interacts with both KU and DNA-PKcs, it has been suggested that it might act as a scaffold to bridge KU-DNA-PKcs and XRCC4-DNA ligase IV, similar to APLF (Aprataxin-and PNK-like factor) (Rulten et al., 2011; Yuan et al., 2014; Simon et al., 2017). In addition, it has been suggested that RBM14 is a co-activator of RNAP II to enhance the generation of dilncRNAs at DSB sites (Jang et al., 2020).

The heterodimeric SFPQ-NONO complex is another example of how RBPs can promote NHEJ-mediated DSB repair. SFPQ (splicing factor proline/glutamine rich) and NONO (Non-POU domain-containing octamer-binding protein) are both members of the *Drosophila behavior/human splicing* (DBHS) family of proteins. They bind to RNA, DNA and to proteins and have vital roles in mRNA maturation (Knott et al., 2016). SFPQ and NONO also promote various genome maintenance pathways, both as single proteins and as a heterodimer. These pathways include the cellular response to UV lesions, telomere maintenance and DSB repair (Li et al., 2009; Alfano et al., 2016; Deshar et al., 2019; Petti et al., 2019). Depletion of either protein sensitizes cells to ionizing radiation, and DSB formation induces the PARP1-dependent re-localization of SFPQ-NONO to damaged chromatin (Li et al., 2009; Salton et al., 2010; Ha et al., 2011; Krietsch et al., 2012). Interestingly, the recruitment to DSBs is mediated by the RNA recognition motif 1 (RRM1) in NONO, which, similar to FUS, can bind to both RNA and poly (ADP-ribose) chains (Ha et al., 2011; Krietsch et al., 2012). Similar to CIRBP and RBMX, SFPQ-NONO accumulation at DSBs is very rapid and the heterodimer is removed from damaged chromatin within minutes following the DNA insult (Salton et al., 2010; Ha et al., 2011). The RNA-dependent recruitment of SFPQ-NONO appears to be regulated by its interacting protein MATR3 (Matrin 3), whose depletion increases the retention time of the heterodimer on damaged chromatin (Salton et al., 2010). Interestingly, both MATR3 and SFPQ-NONO have been implicated in the retention of defective RNAs in nuclear paraspeckles, raising the possibility that similar regulatory mechanisms govern RNA dynamics at damaged chromatin (Zhang and Carmichael, 2001). At DSBs, SFPQ-NONO interacts with the KU proteins and XRCC4-DNA ligase IV and stimulates DNA end-joining, likely by acting as a scaffold that stabilizes DNA pairing in the XRCC4-DNA ligase IV ligation complex (Udayakumar et al., 2003; Bladen et al., 2005; Ha et al., 2011; Krietsch et al., 2012; Li et al., 2014; Udayakumar and Dynan, 2015; Jaafar et al., 2017). In addition, SFPQ-NONO stimulates auto-phosphorylation of DNA-PKcs, although the molecular mechanism of this stimulation is unknown (Udayakumar and Dynan, 2015).

## RNA-Binding Proteins Promote Double-Strand Break Repair via Homologous Recombination

As is the case for NHEJ, RBPs directly facilitate multiple steps of HR-mediated DSB repair including DNA end-resection, RAD51 filament and D-loop formation. DNA end-resection, which channels the DSB response toward HR during repair pathway choice, is regulated by at least two related but distinct hnRNPs: hnRNP UL1 and hnRNP UL2. hnRNP UL1 (hnRNP U-like 1) participates in mRNA processing and transport and represses basic transcription driven by certain viral and cellular promoters (Gabler et al., 1998; Kzhyshkowska et al., 2003). hnRNP UL2 (hnRNP U-like 2) is related to hnRNP UL1, but much less is known about its function in gene expression and RNA metabolism. Loss of both hnRNP UL1 and hnRNP UL2 sensitizes cells to DSB inducing agents (Polo et al., 2012; Hong et al., 2013). Both proteins are recruited to DSB sites by interacting with the NBS1 subunit of the MRN complex (Polo et al., 2012; Hong et al., 2013). The chromatin association of hnRNP UL1 and UL2 is PARP1-dependent, and the proteins are released from the DSB site within minutes following DNA damage induction. Interestingly, their recruitment is sensitive to RNase A treatment and, in the case of hnRNP UL1, depends on the presence of its C-terminal RNA-binding domain, suggesting that local RNA binding contributes to the association of these proteins with chromatin (Polo et al., 2012; Hong et al., 2013). At DSB sites, hnRNP UL1, and UL2 mediate HR-directed repair by promoting DNA end resection downstream of MRN and CtIP, likely by stimulating BLM recruitment to chromatin (Polo et al., 2012).

Besides DNA end-resection, RBPs can also promote HR-directed repair by participating in steps that are further downstream in the repair pathway. An example of such an RBP is FUS, which not only binds RNA and poly (ADP-ribose) chains but also to single- and double-stranded DNA. Although *in vivo* data on a role for FUS in HR is still lacking, it is able to stimulate D-loop formation *in vitro*, suggesting a possible direct role in strand exchange during HR, in addition to its functions in DSB signaling and NHEJ (Crozat et al., 1993; Zinszner et al., 1997; Baechtold et al., 1999; Bertrand et al., 1999). However, further studies are required to confirm that FUS does indeed directly participate in HR-mediated DSB repair.

A second example is hnRNP C, which is a tetrameric protein complex that is formed by the two isoforms hnRNP C1 and hnRNP C2. It is a core component of 40S ribonucleoprotein particles, which regulate the splicing, stability and nuclear export of mRNAs (Huang et al., 1994). After DSB induction, hnRNP C associates with damaged chromatin (Lee et al., 2005; Anantha et al., 2013). As is the case with the hnRNP UL proteins, this association is sensitive to RNase A treatment, suggesting that the recruitment of hnRNP C to DSBs is RNA-dependent (Anantha et al., 2013). However, which type of RNA mediates hnRNP C or hnRNP UL recruitment is not known. Loss of hnRNP C reduces HR-mediated DSB repair and upregulates alternative NHEJ, indicating a pro-HR role in DSB repair pathway choice (Anantha et al., 2013). The molecular details by which hnRNP C participates in HR remain unclear. However, its repair function at damaged chromatin appears to be driven by the RNA-dependent association with PALB2-BRCA1/2 complexes, which mediate DSB pathway choice and also RAD51 loading (Anantha et al., 2013). Of note, similar to FUS, hnRNP C might also directly participate in NHEJ, since it binds to KU and is phosphorylated by DNA-PKcs, although both the significance of these interactions for NHEJ and the mechanistic details remain unclear (Zhang et al., 2004).

The spliceosome component GEMIN2 (Gem-associated protein 2) has also been demonstrated to promote HR at the level of RAD51 loading and strand exchange (Takizawa et al., 2010; Takaku et al., 2011; Sarachan et al., 2012). GEMIN2 is a component of the SMN complex, which is essential for the assembly of spliceosomal small nuclear ribonucleoproteins (snRNPs) (Zhang et al., 2008). GEMIN2 interacts directly with the SMN protein (survival motor neuron), and this interaction is not only relevant for its function in mRNA splicing but also for its role in HR repair. Indeed, SMN-GEMIN2 binds directly to DNA and RAD51 and supports RAD51 filament formation, RAD51-mediated homologous pairing and DNA strand exchange (Takizawa et al., 2010; Takaku et al., 2011; Sarachan et al., 2012).

Finally, in addition to its function during NHEJ, SFPQ also promotes HR-directed DSB repair. The HR function of SFPQ does not occur in complex with NONO but is instead dependent on distinct interactions with the RAD51 recombinase and the RAD51-paralog RAD51D (Morozumi et al., 2009; Rajesh et al., 2011). Besides RNA, SFPQ has previously been shown to bind to both single-stranded and double-stranded DNA and to promote single-strand DNA annealing and the formation of D-loop structures *in vitro*, which closely resemble HR intermediates (Akhmedov and Lopez, 2000). *In vivo*, SFPQ additionally binds to the ATPase domain of RAD51 via its N-terminal PSF domain, which activates RAD51-mediated homology search and strand exchange, particularly early in the HR process when RAD51 accumulation at DSB sites is still low (Morozumi et al., 2009). The mechanistic signficance of the interaction with RAD51D remains unclear, although it has been speculated that it might regulate the RAD51-paralog complex BCDX2 (RAD51B, RAD51C, RAD51D, XRCC2), which facilitates the assembly and stability of the RAD51 nucleoprotein filament during HR (Masson et al., 2001; Rajesh et al., 2011; Chun et al., 2013).

Finally, numerous additional RBPs promote DSB repair by regulating the generation and processing of various RNA species and of RNA:DNA heteroduplex structures that form locally around the break site. These RBPs will be discussed in the next section.

## RNA-Binding Protein-RNA Interactions are Central to Double-Strand Break Signaling and Repair

While some DSB-associated RBPs have roles that are RNA-independent and are based on protein-protein interactions, many RBP functions in this DNA damage pathway are intimately linked to those of their associated RNAs. Although canonical transcription is silenced in an ATM-dependent manner around DSBs, RBPs bind to a wide variety of non-coding RNAs at break sites, including long noncoding RNAs (lncRNAs) and small noncoding RNAs (sncRNAs) such as microRNAs and DNA damage response small RNAs (DDRNAs) (Kruhlak et al., 2007; Shanbhag et al., 2010; Francia et al., 2012; Pankotai et al., 2012; Wei et al., 2012; Morales et al., 2016; Michelini et al., 2017; Thapar, 2018; Ketley and Gullerova, 2020). These noncoding RNAs promote the DSB response by helping to recruit DSB signaling and repair factors to chromatin, either directly through RNA-protein interactions or indirectly by contributing to phase separation locally around the break site. In addition, they can assist repair by temporarily stabilizing the DSB ends, protecting the 3′ ssDNA overhangs from nucleases during end resection, modulating DNA-protein interactions and protein activities, by serving as repair templates and by regulating local chromatin remodeling (Storici et al., 2007; Shen et al., 2011; Wei et al., 2012, 2015; Sharma and Misteli, 2013; D’ Adda di Fagagna, 2014; Keskin et al., 2014; Yang and Qi, 2015; Chakraborty et al., 2016; Thapar, 2018; Durut and Mittelsten Scheid, 2019; Bader et al., 2020; Ketley and Gullerova, 2020; Liu et al., 2021).

From the point of view of DSB-associated RBPs, non-coding RNAs often help to integrate these RBPs into the local signaling and repair network. For example, as is the case for SFPQ-NONO and hnRNP C, non-coding RNAs can act as molecular scaffolds that help to recruit RBPs to damaged chromatin (Ha et al., 2011; Krietsch et al., 2012; Anantha et al., 2013). While the exact identities of these scaffold RNAs are mostly unknown, one well-characterized RNA that has been proposed to act as a scaffold for DNA damage response-relevant RBPs is the lncRNA *NORAD* (non-coding RNA activated by DNA damage). *NORAD* is upregulated in response to DNA damage and interacts with at least 41 distinct RBPs, including SFPQ, RBMX, and PRPF19 (Lee et al., 2016; Munschauer et al., 2018). Another lncRNA that is induced in a DNA damage-dependent manner and that assists in protein recruitment through RNA-protein interactions is *DDSR1* (DNA damage sensitive RNA 1) (Sharma et al., 2015). *DDSR1* binds directly to both BRCA1 and hnRNP UL1, and its loss impairs the chromatin recruitment of BRCA1-RAP80. It has been proposed that *DDSR1* acts in complex with hnRNP UL1 to enhance BRCA1 recruitment and as such promotes HR-directed DSB repair (Sharma et al., 2015).

In addition to RBP recruitment, RNAs also modulate the activity of DSB-associated RBPs. Indeed, numerous lncRNAs have been shown to directly bind to SWI/SNF chromatin remodeling complexes to modulate their activities in response to DNA damage (Prensner et al., 2013; Cajigas et al., 2015; Adriaens et al., 2016). In addition, KU and DNA-PKcs are themselves RBPs and their RNA-binding activity is important for efficient NHEJ-mediated DSB repair (Yoo and Dynan, 1998; Baltz et al., 2012; Britton et al., 2013; Thapar et al., 2020). An RNA that is especially relevant in this context is *LINP1* (lncRNA in nonhomologous end joining pathway 1), which directly binds to KU80 and stabilizes the KU-DNA-PKcs complex at DSB ends (Zhang et al., 2016; Wang X. et al., 2018).

**FIGURE 3:**
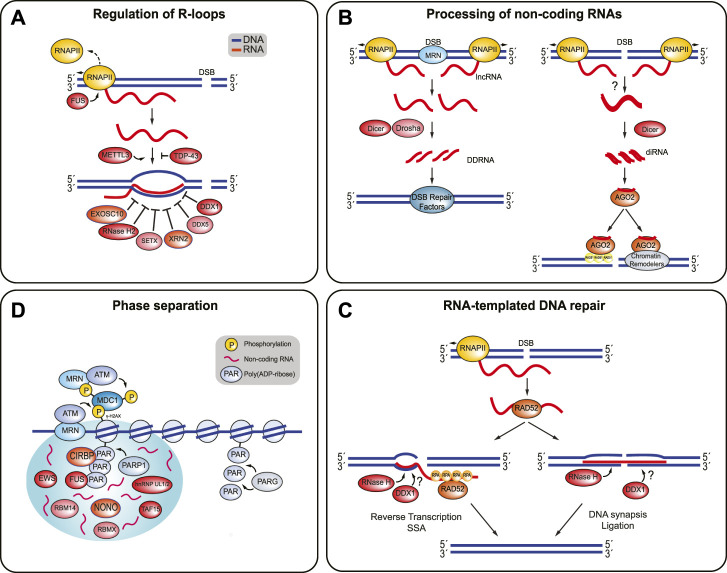
RBPs involved in the regulation of R-loop structures, processing of non-coding RNAs, RNA-templated DNA repair, and phase separation. **(A)** The presence of R-loops at DSBs is highly regulated. FUS prevents R-loop formation by promoting RNAP II dissociation. TDP-43 prevents the nascent RNA from binding to DNA. METTL3 stabilizes these RNA:DNA hybrids. EXOSC10, RNase H2, SETX, XRN2, DDX5, and DDX1 are some of the RBPs involved in resolving RNA:DNA hybrids and R-loops. **(B)** Non-coding RNAs can be processed into DDRNAs or diRNAs. DDRNAs are produced when MRN complex induces production of dilncRNAs by recruiting RNAP II to both ends of the DSB break. The dilncRNAs can be processed by Drosha and Dicer into DDRNAs, which recruit DSB repair proteins to the DSB. In addition, diRNAs are created when RNA transcripts are synthesized near a DSB site by RNAP II become double-stranded and are processed by Dicer. It is unclear how the RNA becomes double-stranded in humans. The diRNAs are then incorporated into Argonaute-2 (AGO2) and AGO2 can then recruit RAD51. AGO2 also helps localize chromatin modifiers to the break site, which also promotes HR. **(C)** RNA-templated DSB repair begins with RAD52 binding to a ssRNA transcript and facilitating RNA:DNA hybrid formation. RAD52 can stimulate DNA recombination or end joining. DNA recombination is promoted when RAD52 undergoes inverse strand exchange, with help from RPA. Reverse transcription and SSA annealing completes the repair process. On the other hand, RAD52 promotes end joining by bridging both ends of the DSB with the homologous RNA transcript. DNA synapsis and ligation finish the repair. The RNA transcript is most likely removed from the DNA by RNase H enzymes and possibly DDX1. **(D)** PARP1 attaches poly (ADP-ribose) (PAR) chains to the DNA surrounding the DSB. Several RBPs are recruited to the DSB site in a PARP-1 dependent manner, where they may contribute promote phase separation around the break site. Non-coding RNAs are likely present and encourage phase separation. PARG disrupts phase separation and releases the RBPs by hydrolyzing the PAR chains.

DSB-associated RBPs often bind to RNAs to prevent the formation of or to clear RNA-associated impediments to DSB repair. Such impediments may appear in the form of RNA:RNA and RNA:DNA hybrids, or as so-called R-loops, which are three-stranded structures consisting of a RNA:DNA hybrid and a displaced strand of DNA (Figure 3A). R-loops tend to form behind RNA polymerases during transcription when the nascent RNA transcript binds to the complementary template DNA (Hegazy et al., 2020). Various RNA-binding helicases and also RNases remove and degrade RNAs at DSBs. For example, depletion of either EXOSC10 (exosome component 10), SETX (Senataxin), or RNase H2 leads to an increase in RNA:DNA hybrids and to defective DNA repair (Mischo et al., 2011; Skourti-Stathaki et al., 2011; Richard et al., 2013; Amon and Koshland, 2016; Lafuente-Barquero et al., 2017; Domingo-Prim et al., 2019; Zatreanu et al., 2019). They likely represent alternative mechanisms to clear RNA:RNA hybrids that are generated by dilncRNAs at DSB sites. DilncRNAs are transcribed from and toward DNA ends whenever RNAP II binds to break-associated MRN (Figure 3B) (Michelini et al., 2017; Sharma et al., 2021). They have important roles in the recruitment of DSB signaling factors and also serve as pre-cursors for the Drosha and Dicer-dependent generation of DNA damage response small RNAs (DDRNAs) (Francia et al., 2012; Michelini et al., 2017; Rossiello et al., 2017; Gioia et al., 2019; Rzeszutek and Betlej, 2020).

At DSBs, RNAP II also promotes the generation of DSB-induced RNAs (diRNAs), which are produced when local RNA transcripts become double-stranded and are then processed by Dicer into double-stranded small RNAs (Wei et al., 2012; Yang and Qi, 2015; Rzeszutek and Betlej, 2020). In *Arabidopsis thaliana*, the generation of double-stranded RNAs depends on RNA-dependent RNA polymerases that use RNA transcripts as templates (Wei et al., 2012; Miki et al., 2017; Rzeszutek and Betlej, 2020). However, how single-stranded RNA transcripts become double-stranded RNAs in human cells remains unclear. Regardless, once formed, DSB-dependent diRNAs are incorporated into Argonaute-2 (AGO2) and guide this protein to damaged chromatin (Gao et al., 2014). Here, AGO2 helps to recruit RAD51 to resected DNA and also acts as a scaffold for chromatin modifiers such as MMSET and Tip60 (Gao et al., 2014; Wang and Goldstein, 2016).

Like RNAP II, RNA Polymerase III (RNAP III) is also recruited to DSBs in a MRN-dependent manner (Liu et al., 2021). After MRN and CtIP begin end-resection, RNAP III uses the resulting ssDNA region to synthesize RNA and displace the 5′ strand, which generates a RNA:DNA hybrid. These RNA:DNA hybrids likely promote HR by preventing the 3′ ssDNA overhang that is essential for RAD51-mediated strand exchange from being degraded during end-resection. Accordingly, depletion of RNAP III in mammalian cells hinders DNA end-resection and HR-directed repair (Liu et al., 2021). It is unclear which proteins resolve RNA:DNA hybrids produced by RNAPIII, but it is possible that SETX, XRN2, EXOSC10, RNase H2, and/or DDX1 are involved in their processing since all of these RBPs promote HR-mediated DSB repair (Marin-Vicente et al., 2015; Li et al., 2016; Cohen et al., 2018; D’Alessandro et al., 2018; Dang and Morales, 2020).

The RNA-binding protein SETX localizes to DSBs at transcribed loci and, together with the 5′-3′ exoribonuclease XRN2 (exoribonuclease 2), unwinds RNA:DNA hybrids (Kim et al., 1999; Skourti-Stathaki et al., 2011; Martin-Tumasz and Brow, 2015; Morales et al., 2016; Groh et al., 2017; Cohen et al., 2018). SETX likely cooperates with the EXOSC10-containing RNA exosome complex to unwind and degrade dilncRNAs that hybridize at DSBs (Richard et al., 2013; Domingo-Prim et al., 2019). Interestingly, EXOSC10 has been shown to be important for the recruitment of RAD51 to DSBs, suggesting that the clearance of dilncRNA-associated RNA:RNA and RNA:DNA hybrids is required for effective HR (Marin-Vicente et al., 2015). RNase H2 removes dilncRNAs from DSB sites independent of SETX and EXOSC10 (Domingo-Prim et al., 2020). Another RNA helicase active at DSBs is DEAD Box 1 (DDX1), which unwinds both RNA:RNA and RNA:DNA hybrids (Chen et al., 2002; Li et al., 2008; Li et al., 2016). DDX1 accumulates at DSBs in an ATM- and 53BP1-dependent manner (Li et al., 2008; Li et al., 2017). At break sites, DDX1 removes DDRNA-induced RNA:DNA hybrids from ssDNA after DNA end-resection (Li et al., 2016, 2017). In addition, DDX1 has been proposed to promote HR-directed repair by interacting with and recruiting the BLM helicase to damaged chromatin (Li et al., 2008; Li et al., 2017).

DDX1 has also been implicated in RNA-mediated DSB repair, which uses single-stranded (ss) RNA transcripts to coordinate repair (Figure 3C) (Storici et al., 2007; Shen et al., 2011; Keskin et al., 2014; Wei et al., 2015; Li et al., 2017). RNA-mediated DSB repair critically depends on the RNA-binding protein RAD52, which facilitates the formation of a DNA:RNA heteroduplex that favors DSB repair, either by promoting the annealing of an RNA transcript to resected ssDNA or by catalyzing the inverse strand exchange between an RNA transcript and homologous dsDNA (Keskin et al., 2014; Mazina et al., 2017; McDevitt et al., 2018). RAD52 promotes RNA-mediated DSB repair via two possible mechanisms, which lead to fundamentally different repair outcomes by promoting either DNA end-joining or DNA recombination. First, RAD52 facilitates DSB end bridging by mediating the annealing of a homologous RNA transcript with two adjacent blunt or partially resected DNA ends. The formation of this RNA:DNA heteroduplex then favors DNA synapsis and DNA ligation (Chakraborty et al., 2016; Mazina et al., 2017; McDevitt et al., 2018). Second, RAD52 promotes the partial annealing of a homologous RNA transcript with either a resected DSB or a blunt DSB end (via inverse strand exchange) to initiate reverse transcription using the RNA as a template. The reverse transcription is then followed by repair completion via single-strand annealing (Mazina et al., 2017; McDevitt et al., 2018). Interestingly, RNA-templated DSB repair is stimulated by RPA, which is able to bind to RAD52 but also to ssRNA, albeit with lesser affinity than for ssDNA (Kim et al., 1992; Mazina et al., 2017). Although the mechanistic details remain to be fleshed out, it has been proposed that the RNA-binding activity of RPA induces a conformational change in RAD52 that favors strand exchange between DSB ends and homologous RNA templates (Keskin et al., 2014; Mazina et al., 2017). In budding yeast, translesion DNA polymerase *ζ* is able to reverse transcribe RNA and use it as a repair template at DSB sites (Meers et al., 2020). It will be important to test whether human Pol *ζ* or other human DNA polymerases similarly promote reverse transcription to fill in gaps during RNA-templated DNA repair. Finally, increasing the stability of the RNA:DNA hybrid through depletion of RNase H1 and RNAse H2 was shown to increase the frequency of RNA-templated DNA repair events (Keskin et al., 2014; Mazina et al., 2017). How DDX1 contributes to RNA-dependent DSB repair is not clear, but it might be related to its function in clearing RNA:DNA hybrids that would otherwise impede strand exchange. Like other RNA:DNA hybrid structures, R-loops pose considerable roadblocks for the DSB repair machinery. Numerous DSB-associated RBPs, including TDP-43, FUS, SFPQ, and NONO, have been reported to prevent the formation of R-loops and RNA:DNA hybrids (Hill et al., 2016; Wang I. X. et al., 2018; Petti et al., 2019; Gianini et al., 2020; Jang et al., 2020; Wood et al., 2020). For example, TDP-43 binds to nascent RNA and precludes the RNA from binding to the complementary DNA template strand (Wood et al., 2020). In contrast, FUS directly interacts with active RNAP II and facilitates RNAP II dissociation. As such, it prevents R-loop formation by avoiding excessive RNAP II-mediated transcription at DSB sites (Schwartz et al., 2012; Kwon et al., 2013; Hill et al., 2016). DDX5, a DEAD box-containing RNA helicase like DDX1, also unwinds RNA:DNA hybrids and R-loops at DSB sites (Xing et al., 2017; Mersaoui et al., 2019; Yu et al., 2020; Sessa et al., 2021). It binds to RNA that is transcribed *in cis* to a DSB to remove RNA-associated impediments and enable HR-directed repair (Yu et al., 2020; Sessa et al., 2021). Accordingly, loss of DDX5 results in the accumulation of polarized end deletions that specifically occur on those sides of DSBs that are actively transcribed and as such generate RNA species that can hinder the HR machinery (Yu et al., 2020). DDX5 interacts directly with the HR mediator BRCA2, which helps to enrich DDX5 at DSB sites, and which stimulates its RNA helicase activity *in vitro* (Sessa et al., 2021). Interestingly, like numerous other RBPs, DDX5 is then rapidly excluded again from damaged chromatin (Salton et al., 2010; Ha et al., 2011; Adamson et al., 2012; Polo et al., 2012; Britton et al., 2014; Altmeyer et al., 2015; Chen et al., 2018; Yu et al., 2020; Sessa et al., 2021). This exclusion is ATM- and transcription-dependent and requires the RNA-binding domain in DDX5 (Yu et al., 2020). Finally, the ribonucleases RNase H1 and RNase H2 are essential for resolving R-loops during replication (Cerritelli and Crouch, 2009). It is unclear, whether they are also involved in the clearance of DSB-associated R-loops (Zhao et al., 2018). However, given the role of RNase H2 in removing dilncRNAs, a similar function in R-loop processing at DSBs seems likely (D’Alessandro et al., 2018).

## The Role of RNA-Binding Protein-Mediated Phase Separation at Double-Strand Break Sites

It is becoming increasingly evident that liquid-liquid phase separation (LLPS) around damaged chromatin is necessary for efficient DNA damage signaling and repair, and DSB-associated RBPs appear to be important drivers of phase separation around DSB sites (Kilic et al., 2019; Pessina et al., 2019, 2020). LLPS is a reversible process in which a solution containing proteins and/or nucleic acids is converted into two liquid phases, a dense phase and a dilute phase. These condensed liquid-like droplets, often referred to as biomolecular condensates or membrane-less organelles, are maintained through dynamic interactions and weak intermolecular bonds between the molecules within the droplet (Hyman et al., 2014; Pessina et al., 2020). *In vivo*, membrane-less organelles are formed by a combination of RNAs that interact with proteins containing intrinsically disordered regions, which in many have RNA-binding properties. LLPS dynamics within these organelles are influenced by the RNA to protein ratio, and by post-translational modifications such as phosphorylation, acetylation, and SUMOylation, which can promote or inhibit LLPS by strengthening or weakening molecular interactions of the phase-separated proteins (Hyman et al., 2014; Molliex et al., 2015; Patel et al., 2015; Maharana et al., 2018; Hofweber and Dormann, 2019). Examples of membrane-less organelles formed through LLPS are Cajal bodies, paraspeckles, stress granules and nucleoli (Pessina et al., 2020). Intriguingly, DNA repair foci, which are formed around damaged chromatin and contain DSB signaling and repair factors, are viscous yet highly dynamic structures that are sensitive to agents that disrupt liquid-like droplets, suggesting that they, too, can be regarded as LLPS-induced membrane-less organelles (Pessina et al., 2019; Pessina et al., 2020). It has been proposed that LLPS enables an efficient DSB response by concentrating DNA signaling and repair factors around the break site. Although the molecular details remain to be determined, one model proposes that, PARP is immediately recruited to DSB sites, where it adds poly (ADP-ribose) chains to the DNA surrounding the break site (Figure 3D). The negatively charged, low complexity, and RNA-like poly (ADP-ribose) chains then act as bait for phase-separating proteins with positively charged, intrinsically disordered regions (Altmeyer et al., 2015; Kai, 2016; Pessina et al., 2020). Indeed, many DSB-associated RBPs contain intrinsically disordered regions and they are recruited to DSBs in a PARP-dependent manner, suggesting that they contribute DNA damage-induced local phase separation. These RBPs include FUS, CIRBP, hnRNP UL1, NONO, RBM14, RBMX, TAF15, and EWS (Krietsch et al., 2012; Hong et al., 2013; Mastrocola et al., 2013; Altmeyer et al., 2015; Izhar et al., 2015; Chen et al., 2018). According to the poly (ADP-ribose) chain-dependent phase separation model, these RBPs promote phase separation (and, as discussed above, carry out additional roles in the local DSB response) until the poly (ADP-ribose) chains are hydrolyzed by PARG (poly (ADP-ribose) glycohydrolase), which disrupts LLPS and releases the RBPs from the break site (Illuzzi et al., 2014; Singatulina et al., 2019). In line with this idea is the observation that the clearance of poly (ADP-ribose) chains is important for the recruitment of the downstream DSB signaling factor 53BP1, which itself then participates in RNA-dependent, but poly (ADP-ribose)-independent phase separation at the DSB (Kilic et al., 2019; Pessina et al., 2019). Besides poly (ADP-ribose) chains, non-coding RNAs likely also promote LLPS at DSBs. Indeed, RNase A, RNAP II inhibitors, and sequence-specific antisense oligonucleotide treatments all inhibit DNA repair focus formation, suggesting that the local generation of non-coding RNAs is required for phase separation at DSB sites (Pryde et al., 2005; Francia et al., 2012; Michelini et al., 2017; Rossiello et al., 2017). It is likely that at least a subset of these RNA species are bound by phase-separating RBPs to promote LLPS and to locally retain signaling and repair factors (Pessina et al., 2019).

Of note, many RBPs that participate in DSB signaling and repair have also been identified as phase separation-related proteins in the context of other membrane-less organelles in the nucleus, particularly paraspeckles (Hennig et al., 2015; You et al., 2020). Paraspeckles are subnuclear bodies that contain the lncRNA *NEAT1* and a large variety of proteins, including the DSB-associated RBPs SFPQ, NONO, RBM14, FUS, CIRBP, hnRNP UL1, RBMX, and TDP-43 (Fox et al., 2002; Fox et al., 2018; Naganuma et al., 2012; Nishimoto et al., 2013). They play important roles in gene regulation, the sequestration of proteins and RNAs, and in microRNA processing (Fox et al., 2018; Taiana et al., 2020). Interestingly, paraspeckles have also been linked to the DSB response since many of their core components are involved in DNA repair pathways, and *NEAT1* transcription is enhanced upon DNA damage induction (Blume et al., 2015; Adriaens et al., 2016). Within paraspeckles, *NEAT1* binds directly to the phase-separating proteins SFPQ, NONO, RBM14, and FUS, and its scaffolding function might also be relevant for phase separation in the context of the DSB response (Chen and Carmichael, 2009; Clemson et al., 2009; Sasaki et al., 2009; Sunwoo et al., 2009; Taiana et al., 2020).

## Final Remarks

The cellular response to DSBs is a highly complex DNA damage response pathway, and its coordination, both in space and time, requires multiple layers of regulation. RBPs have long been known to participate in the global DSB response, particularly by helping to adjust gene expression in the face of DNA damage. However, a flurry of recent discoveries has placed RBPs also at the heart of the DSB response that occurs locally on and around the chromatin flanking DSBs (Table 1).

Because RBPs are highly versatile proteins that are able to mediate not only RNA-protein interactions but are also frequently involved in protein-DNA and protein-protein interactions, they are perfectly placed to regulate the intricate interplay of DNA, RNA and signaling and repair proteins that occurs at the DSB site. Indeed, given their molecular versatility, it is not surprising that DSB-associated RBPs touch upon almost every aspect of the chromatin-based DSB response, including chromatin remodeling around the break site, recruitment and regulation of other DSB signaling and repair factors, local RNA metabolism and phase separation (Figure 2). In addition to the many DSB-associated RBPs with classical RNA-binding domains, several well-known DSB response proteins previously not considered to be RNA-binding proteins do indeed bind to RNA (i.e., 53BP1, BRCA1, KU proteins, RPA, and RAD52), although in many cases the significance of these interactions is only poorly understood (Kim et al., 1992; Yoo and Dynan, 1998; Pryde et al., 2005; Keskin et al., 2014; Sharma et al., 2015). It will be important to determine whether and how the RNA-binding activities of these unconventional RBPs contribute to their functions in DSB signaling and repair.

To date, details of the molecular mechanisms of action of most other DSB-associated RBPs are similarly lacking. For example, while RNA-RBP interactions are clearly important for timely and efficient DSB repair, the DNA damage-dependent RNA interactome of many DSB-associated RBPs remains uncharacterized. The identification of these RNAs has the potential to shed much needed light onto the mechanisms underlying the recruitment and function of RBPs at DSBs. Of note, most studies have so far focused on the effects of RBPs on the generation and function of various RNA species at DNA damage sites. However, whether and how RNAs in turn modulate protein functions in the context of the chromatin-based DSB response has not been addressed. A related question pertains to the specificity of RBP recruitment and function at DSB-flanking chromatin. Despite the fact that cells contain well over 1,500 RBPs, many of which play key roles in mRNA processing, splicing and transport and are, by nature, promiscuous in terms of RNA-binding activity, the RNA-binding proteome at damaged chromatin is nevertheless specific and appears to be tightly regulated (Gerstberger et al., 2014). A RBP family that is enriched at DSBs are hnRNPs, which are best known for their diverse functions in transcription, splicing, mRNA stabilization and translational regulation (Geuens et al., 2016). This family contains around 30 members, a third of which have been implicated in the DSB response (Table 1). hnRNPs are classical RBPs that achieve RNA-binding specificity and plasticity mainly through the combinatorial use of several types of RNA-binding domains (Geuens et al., 2016). However, these properties also guide many of their known functions in mRNA metabolism and cannot alone explain the mechanisms underlying their targeted accumulation at damaged chromatin. It seems likely that the specificity of hnRNPs and of other RNA-binding proteins for DSB sites is achieved by the recognition of local RNA targets combined with DSB-specific protein-protein interactions and DNA damage-induced post-translational protein and possibly even RNA modifications.

In the same vein, the chromatin association of most RBPs is highly dynamic and, in a number of cases, results in the rapid dissociation and even complete exclusion from DSB sites (Salton et al., 2010; Ha et al., 2011; Adamson et al., 2012; Beli et al., 2012; Britton et al., 2014; Altmeyer et al., 2015; Izhar et al., 2015; Chen et al., 2018; Yu et al., 2020; Sessa et al., 2021). While the exact molecular purposes and the underlying regulatory mechanisms of this behavior remain largely mysterious, it is attractive to speculate that they might be, at least in part, linked to the LLPS process, which by nature, needs to be dynamic, fluid and adaptive. Removing RBPs and their associated RNAs at certain times post DNA damage induction may adjust the properties of the local LLPS organelle to facilitate downstream signaling or repair reactions. An additional, not mutually exclusive, possibility is that the exclusion of certain RBPs results from the positive and negative transcription dynamics that occur around DSB sites, and which are tightly linked to the generation and processing of various RNA species, RNA:DNA hybrids and R-loops (Shanbhag et al., 2010; Polo et al., 2012; Britton et al., 2014). A case in point supporting this idea is DDX5, whose exclusion from damaged chromatin is dependent on RNAP II-mediated transcription at the break site (Yu et al., 2020). Why DDX5 needs to be removed from damaged chromatin as soon as it clears RNA-associated DNA repair impediments is not clear, but it might be related to the observation that not all RNA:DNA hybrids are detrimental to the repair process. Instead, the context of their formation seems to determine whether they need to be removed or stabilized (Marnef and Legube, 2021). A consequence of this RNA:DNA hybrid plasticity is that the activity of the RBPs that control the processing of these structures needs to be tightly regulated, and the exclusion of these proteins at certain repair steps might be a reflection of this regulation. DDX5 is only one of numerous RBPs that are involved in the processing of RNA:DNA duplexes and R-loops at DSBs (Table 1). Intriguingly, many of these RBPs have opposing activities, particularly with regards to the stabilization or resolution of these structures. For example, while RBM14 and METTL3 stabilize RNA:DNA hybrids to promote DSB repair, DDX5, DDX1, SETX, and many other RBPs instead mediate their resolution (Li et al., 2008, 2016; Cohen et al., 2018; Jang et al., 2020; Yu et al., 2020; Zhang et al., 2020; Sessa et al., 2021). This dichotomy of RBP functions with regards to RNA:DNA hybrids further supports the idea that the context and timing of these structures is critical for efficient DSB repair (Marnef and Legube, 2021). Determining how RNA:DNA hybrids promote certain DSB repair steps while inhibiting others, and how this is regulated by RBPs, are important open questions.

Finally, genome instability contributes to the aging process and is also a key driver of many diseases (Tubbs and Nussenzweig, 2017; Alt and Schwer, 2018; da Silva and Schumacher, 2021). Intriguingly, many of the DSB-associated RBPs discussed here have been linked to the development of cancer and also to the onset of neurodegeneration (Castello et al., 2013; Geuens et al., 2016; Low et al., 2020). Prime examples of RBPs involved in neurodegeneration are FUS and TDP-43, both of which play important roles in the etiology of ALS and other brain disorders (Deng et al., 2014; Gao et al., 2018). These diseases are characterized by the aberrant aggregation of RBPs (and other proteins) and also by genome instability. Despite extensive research efforts, the molecular details underlying their pathology are not completely understood. However, it is becoming clear that the protein aggregation and genome instability phenotypes are interconnected, at least in certain settings, and that defects in LLPS might be their common denominator (Alberti and Dormann, 2019; Pessina et al., 2020; Sun et al., 2020). Major questions that need to be addressed now are how exactly aberrant phase separation affects RBP-mediated genome maintenance in neurons and how this contributes to the genome instability and to the protein aggregates observed in the brains of neurodegeneration patients. Similar questions arise also beyond neurodegeneration as RBPs and genome instability have firm connections to other diseases as well, particularly to cancer. Determining how DSB-associated RBPs contribute to genome maintenance, how their dysfunction in the global and chromatin-based DSB response drives disease, and whether they can provide rationales for new treatment approaches are key challenges that need to be addressed in the future.
